# Associated Pathologies following Luxatio Erecta Humeri: A Retrospective Analysis of 38 Cases

**DOI:** 10.3390/jcm11020453

**Published:** 2022-01-17

**Authors:** Roman C. Ostermann, Julian Joestl, Marcus Hofbauer, Christian Fialka, Jakob E. Schanda, Maximilian Gruber, Harald Binder, Thomas M. Tiefenboeck

**Affiliations:** 1 St. Vincent Shoulder Clinic, Baumgasse 20A, 1030 Vienna, Austria; 2 Department of Orthopedics and Trauma Surgery, Medical University of Vienna, Austria Waehringer Guertel 18-20, 1090 Vienna, Austria; ordination@dr-joestl.at (J.J.); marcus.hofbauer@meduniwien.ac.at (M.H.); maximilian.gruber@t-online.de (M.G.); harald.binder@meduniwien.ac.at (H.B.); thomas.tiefenboeck@meduniwien.ac.at (T.M.T.); 3 AUVA Trauma Center Vienna-Meidling, 1120 Vienna, Austria; christian.fialka@auva.at (C.F.); jakob.schanda@auva.at (J.E.S.)

**Keywords:** luxatio erecta humeri, inferior shoulder dislocation, shoulder dislocation, greater tuberosity fracture, rotator cuff tear, neurologic lesion

## Abstract

Inferior shoulder dislocation in fixed abduction, also known as luxatio erecta humeri (LEH), is a rare injury with little data available. Therefore, the primary aim of this study was to evaluate and present our case series of this type of injury with special emphasis on associated pathologies; the secondary aim was to present diagnostic recommendations to detect for potential associated pathologies typically seen with this injury. A total of 38 patients (13 females, average age 72.8 years and 25 males, average age 41.4 years), who have been treated for inferior shoulder dislocation between 1992 and 2020, were included in this study. Associated pathologies after LEH were found in 81% of the cases. Twenty-one of these patients presented with secondary bony pathologies. Six patients revealed rotator cuff injuries diagnosed by magnetic resonance imaging (MRI). Seven patients exhibited pathological findings at the capsule-ligament complex. Eight patients presented with neurological findings. All neurologic symptoms except one axillary nerve palsy and a radialis paresis dissolved during the follow-up period. Five patients received surgical treatment of the affected shoulder. Inferior shoulder dislocation is a rare condition presenting with a high number of associated injuries. According to the findings of the present study, we want to raised awareness of the high rate of potential secondary shoulder pathologies associated with LEH. Beside a thorough clinical examination and immediate standard radiographs in two planes, we recommend to perform computed tomography scanning and an MRI of the shoulder as soon as possible. In the case of neurologic deficiencies, a determination of nerve conduction should be performed.

## 1. Introduction

With an overall incidence rate ranging from 24 to 56 per 100,000 person-years, the glenohumeral joint reveals the highest dislocation rate of all joints. Anteroinferior shoulder dislocation is the most common form of shoulder dislocation, comprising 95% of all shoulder instabilities [1], whereas inferior shoulder dislocation is a rare condition (0.5%), with the largest series comprising 18 cases and a total of 199 cases reported in the current literature [2]. This condition is also called luxatio erecta humeri (LEH) because the arm appears to be permanently held upward or behind the head, in fixed abduction.

Inferior shoulder dislocations are typically caused by a hyperabduction of the arm that forces the humeral head against the acromion combined with an axillar high-energy vector [3,4]. LEH has a high complication rate of up to 80 percent, comprising fractures of the greater tuberosity, injuries of the rotator cuff or nerve palsy [5,6,7].

To the best of our knowledge, this cohort of 38 cases is the largest ever reported. Therefore, the primary aim of this study was to evaluate and present our case series of patients who suffered from inferior shoulder dislocations with special emphasis on associated pathologies. Additionally, we aimed to develop an algorithm for the diagnosis of potentially associated pathologies following inferior shoulder dislocation.

## 2. Materials and Methods

**Figure 1:**
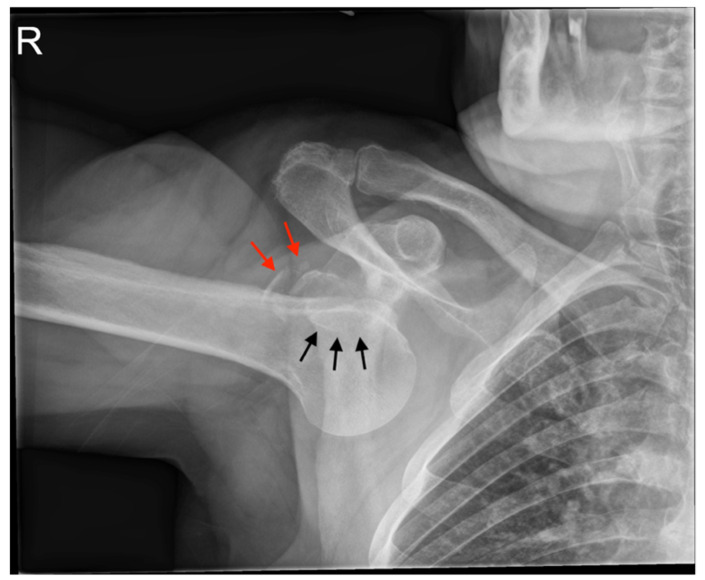
Plain radiography of the right shoulder with inferior dislocation: the arm is fixed in abduction under the inferior glenoid rim (black arrows), the greater tuberosity is fractured (red arrows).

**Figure 2:**
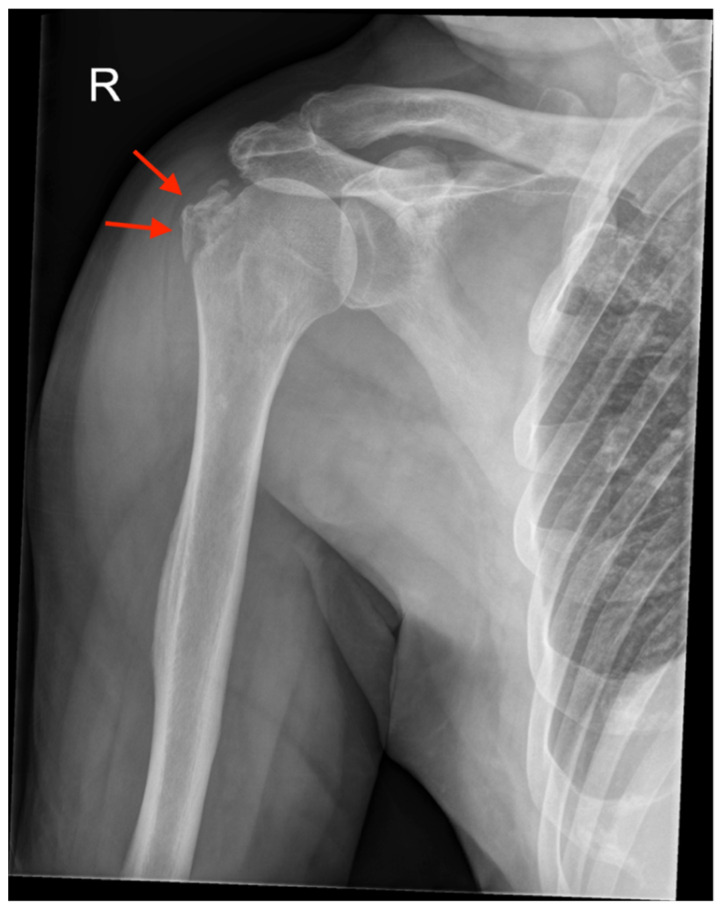
Plain radiography of the right shoulder with inferior dislocation after reduction: the greater tuberosity is fractured and nearly undisplaced after reduction (red arrows).

**Figure 3:**
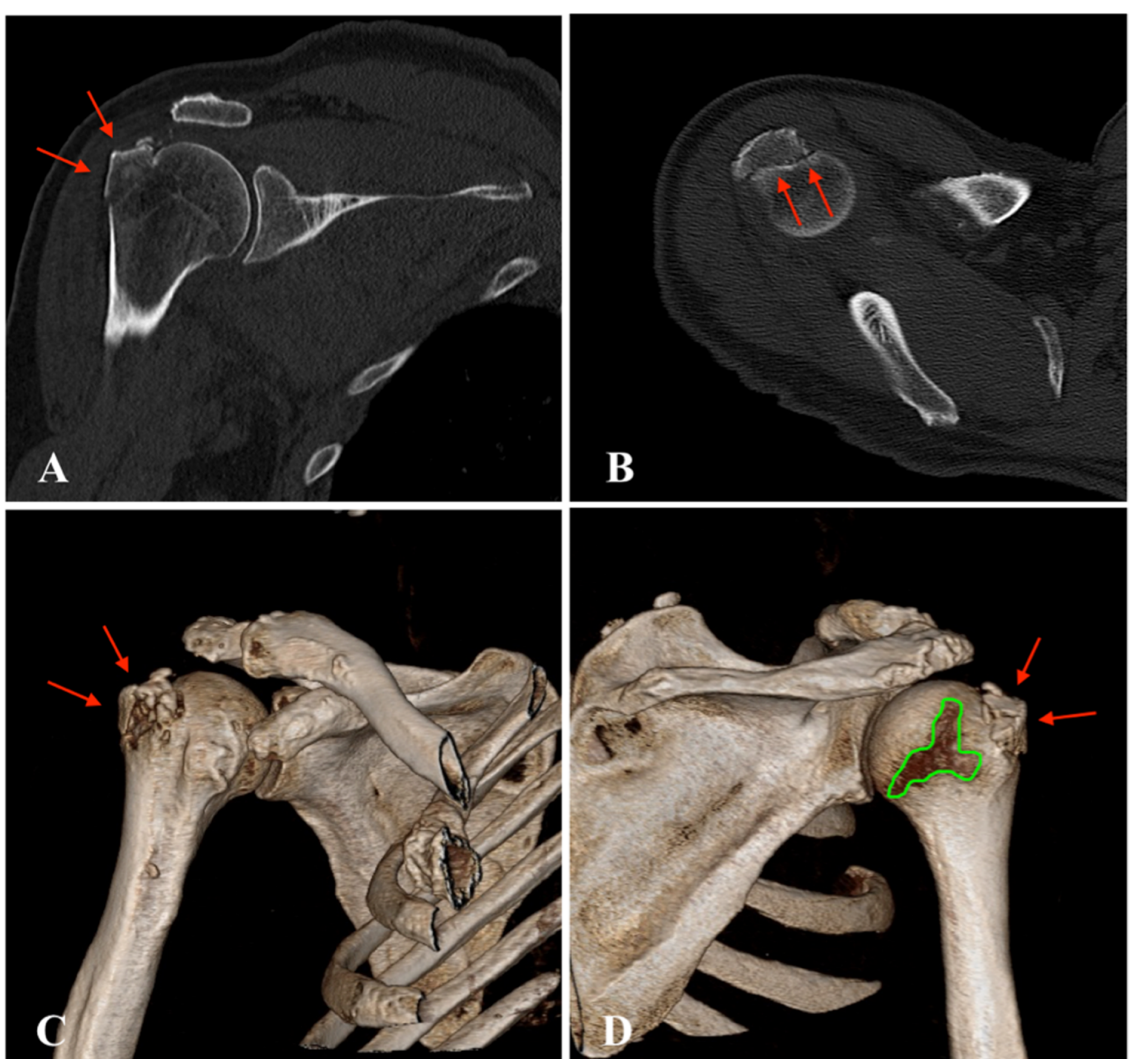
Computed tomography (CT) of the right shoulder after inferior dislocation: (**A**), coronal scan with greater tuberosity fracture (red arrows); (**B**), axial scan with greater tuberosity fracture (red arrows); (**C**), anterior 3D reconstruction with nearly undisplaced greater tuberosity fracture (red arrows); (**D**), posterior 3D reconstruction with nearly undisplaced greater tuberosity fracture (red arrows) and visible bare area of the humeral head (green area).

**Figure 4:**
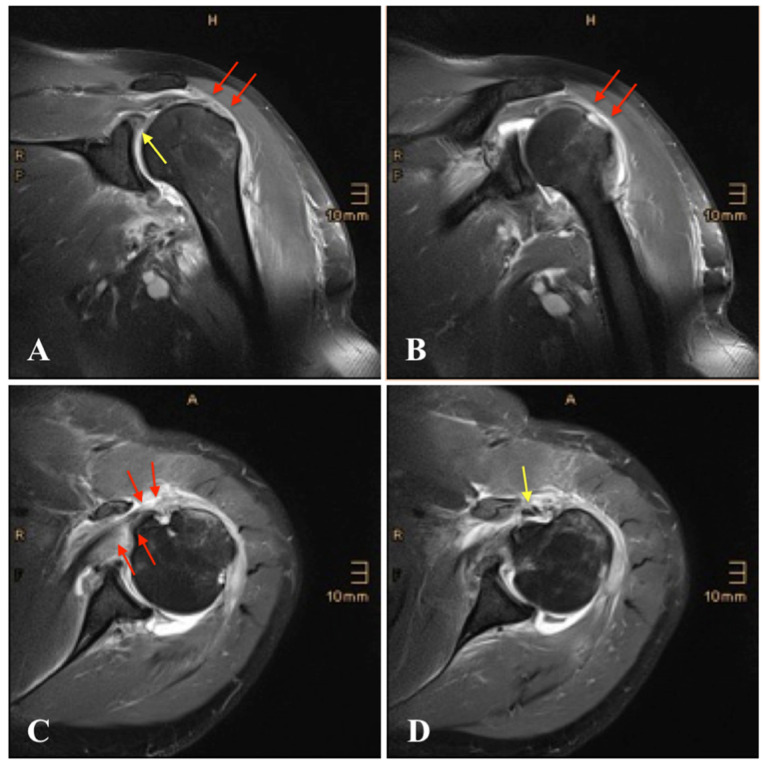
Magnetic resonance imaging (MRI) of the left shoulder following inferior dislocation: (**A**), coronal view with tear of the supraspinatus tendon (red arrows) and superior labrum anterior and posterior (SLAP) lesion (yellow arrow); (**B**), coronal view with tear of the infraspinatus tendon (red arrows); (**C**), axial view with tear of the subscapularis tendon and fatty degeneration of the muscle (red arrows); (**D**), axial view with rupture of the long head of the biceps tendon (LHBT) (yellow arrow).

Archives and databases of the department of trauma surgery of two academic urban Level I trauma centers were systematically screened for the diagnosis of inferior shoulder dislocation. All cases of LEH and documented radiological examination over the past 33 years (1992–2020) were included in this study. Patients with missing radiological documentation of the dislocated state (reduction prior to radiological examination) have been excluded. An experienced radiologist screened all standard radiographs (Figure 1 and Figure 2) and available ultrasound (US), computed tomography (CT) (Figure 3), and magnetic resonance imaging (MRI) (Figure 4) for associated pathologies. Only injuries of the affected shoulder were evaluated; non-shoulder-related pathologies were not addressed.

Due to the small number of patients with different injury patterns, we only performed a descriptive analysis. The measured parameters were analyzed using the SPSS®software (Version 25.0, SPSS Inc., Chicago, IL, USA) and Microsoft®Excel (Version 16.50, Microsoft Inc., Redmont, WA, USA).

## 3. Results

Over the past 33 years, a total of 38 patients, 13 females (mean age 72.8, range 33 to 91 years) and 25 males (mean age 41.4 years, range 16 to 78 years), were referred to the authors’ emergency departments with the diagnosis of inferior shoulder dislocation and met the inclusion criteria. Demographic data including sex, age, affected side, injury pattern, radiologic examination, type of reduction, time of immobilization and follow-up period are shown in Table 1. The mean follow-up period was 3 months (range, 1 to 25 months). Follow-up was defined as the last documented appointment.

Six patients revealed associated injuries to other body regions than the shoulder (cerebral concussion, sprain of cervical vertebrae, thoracic contusion, pertrochanteric femur fracture, patella fracture and knee contusion). Four patients (age 17 to 27 years) presented with previous shoulder dislocations (range 1 to 20 times) of the ipsilateral shoulder. During the documented follow-up of this study, no patient sustained a recurrent dislocation.

All patients underwent standardized radiographic examination of the affected shoulder (Figure 1 and Figure 2). One patient had an US, eight patients a CT scan (Figure 3), nine patients an MRI (Figure 4) and two patients an MR-arthrography (Table 1).

Associated pathologies after LEH were found in 31 patients (82%) and are documented in Table 2.

A total of 23 patients (61%) presented with secondary bony pathologies: two patients sustained a bony Bankart lesion, one patient an anterior labral periosteal sleeve avulsion (ALPSA lesion) with additional glenoid bone loss, two patients a bony avulsion of the inferior glenoid, one patient a bony avulsion of supraspinatus and infraspinatus tendon, five patients a Hill-Sachs lesion, thirteen patients a fracture of the greater tuberosity (Figure 1, Figure 2 and Figure 3) and two patient a proximal humeral head fracture.

Seven patients (18%) exhibited rotator cuff injuries revealed on MRI findings: one bony avulsion of the supraspinatus and infraspinatus tendon, one partial rupture of the supraspinatus and two of the infraspinatus tendon, four ruptures of the supraspinatus (Figure 4), one of the infraspinatus and two of the subscapularis tendon (Figure 4), one total rotator cuff tear, one partial rupture of the long head of the biceps tendon (LHBT) and two complete ruptures of the LHBT (Figure 4).

Additional seven patients were highly suspected to have suffered a rotator cuff tear due to loss of range of motion (ROM) or proximal migration of the humeral head in standard radiographs (Table 2). All patients affirmed that they had full ROM before inferior shoulder dislocation.

Seven patients (18%) exhibited pathological findings at the capsule-ligament complex: two bony Bankart lesions, one ALPSA lesion with additional glenoid bone loss, two bony avulsions of the inferior glenoid, one avulsion of the posterior and inferior labrum and four superior labrum anterior and posterior (SLAP) lesions III. Another patient was highly suspected to have suffered a Bankart lesion due to a diagnosed subluxation of the humeral head seen in standard radiographs (Table 2). However, no further imaging was carried out in that patient.

Eight patients (21%) revealed neurological findings following inferior shoulder dislocation: one patient with ulnar nerve palsy and numbness of the 4th and 5th fingers, one patient with radialis palsy and three patients with axillary nerve palsy, in two cases seen only after reduction of the joint and three patients with mixed nerve palsy with numbness of the 2nd, 3rd and 4th fingers or all fingers, which could not be related to a single nerve. All neurologic symptoms except one axillary nerve palsy and one radialis palsy dissolved during the follow-up period (1 to 11 months) (Table 2).

Six patients (16%) underwent surgical treatment of the affected shoulder (Table 2) at one of the two trauma centers. One patient had an arthroscopic Bankart repair, two patients had an arthroscopic rotator cuff repair accompanied by a subacromial decompression and a biceps tenodesis or tenotomy. One patient had a bony reconstruction of a humeral head impression. Patient number 6, who presented with a total of 20 previous shoulder dislocations, reported no following dislocations during the one-month follow-up. However, in the course of another visit not associated with inferior shoulder dislocation, he reported an additional ten shoulder re-dislocations within the following five years. After an open Latarjet-procedure in another trauma center, the affected shoulder remained stable for over five more years until last. Patient number 27 with a proximal humeral head fracture and dislocation of the humeral head into the axilla with total rupture of the rotator cuff was treated with a primary reverse total shoulder arthroplasty within the first week after inferior shoulder dislocation. Table 3 summarizes the key results of our findings.

## 4. Discussion

Due to the rarity of an LEH injury, comprising of only about 0.5% of all shoulder dislocations 30, data about associated pathologies concerning bone, rotator cuff, capsule-ligament complex or nerves are scarce [3,4,5,6,7]. The presented results of 38 patients with inferior shoulder dislocation injury are, to the best of our knowledge, the largest cohort ever reported.

Current guidelines usually recommend symptomatic patients with anteroinferior shoulder instability to undergo surgical stabilization of the shoulder joint, since it has been proven that stabilization minimizes the risk for recurrent shoulder dislocations [8,9,10]. Due to the high frequency of anteroinferior shoulder dislocations [11,12] and the visualization possible with MRI and during surgery, comprehensive data concerning secondary pathologies has been reported extensively for this type of injury.

In case of traumatic anterior shoulder dislocations, bony defects of the glenoid are reported in 5–56% [13,14] of bony defects of the humerus, in particular Hill-Sachs lesions, in 65–71% of all first-time dislocations and in 93% of recurrent dislocations, respectively [15,16]. In this series of inferior shoulder dislocation, bony defects of the glenoid occurred in 13%, while bony defects of the humerus in 53%. Those rates are slightly higher than the numbers reported by Nambiar et al. [2] but could be explained with the relatively high rate of CT scans and MRI investigations in our series compared to other case series in the literature [2]. Interestingly, only five patients (13%) revealed Hill-Sachs lesions, whereas thirteen (34%) presented with greater tuberosity fractures. Although these percentage rates have to be interpreted with caution due to the small caseload, the occurrence of these secondary pathologies can be explained by the anatomy and the injury pattern. In case of anteroinferior shoulder dislocations, the Hill-Sachs lesion usually occurs due to the impact of the humeral head to the anterior glenoid rim, whereas in the case of inferior shoulder dislocation, the humeral head is positioned somehow beneath the inferior glenoid rim with the arm elastically fixed in abduction. Therefore, greater tuberosity fractures seem to be a more logical consequence of inferior shoulder dislocation and may be the equivalent to Hill-Sachs lesions.

Injuries of the superior rotator cuff following traumatic shoulder dislocations occur in around 30% of patients older than 40 years of age, increasing up to 80% in patients over 60 years of age [17,18,19,20,21,22]. Even younger patients can sustain a rotator cuff rupture during the event of an anteroinferior shoulder dislocation: in the biggest case series investigating isolated subscapularis tendon ruptures, 21% of the tears were caused by shoulder dislocation [23]. In this series, we found rotator cuff tears in seven patients (18%), a rate similar to the one reported by Nambiar et al. in their systematic review [2]. However, including the seven patients with highly suspected rotator cuff tears (restricted ROM, proximal migration of the humeral head in standard radiographs), the rate increases up to 37%. Nevertheless, we cannot rule out a preexisting rotator cuff tear in those seven patients that might have led to the observed superior migration of the humeral head. However, the additionally observed loss of ROM in these seven patients can be seen as a direct cause of the inferior shoulder dislocation, as all patients reported normal ROM of the affected shoulder prior to this injury. The rather high rate of rotator cuff tears could be a consequence of the usually required high-energy vector for inferior shoulder dislocation and the typical position of the humeral head and arm in LEH. Considering the enormous shear forces, it seems likely that the rotator cuff must be at least partially torn while the humeral head with its footprint of the rotator cuff is fixed under the inferior glenoid rim. Similar to the allocation to different age groups in anteroinferior dislocations, rotator cuff tears occurred more often in patients over 50 years of age. Rotator cuff injuries in patients younger than 50 years of age are seen in 8%, whereas in patients over 50 years of age, the percentage of rotator cuff injuries rises up to 63%. Degenerative processes within the tendon, which compromise the sturdiness of muscle and tendon tissue, could be a possible explanation for this difference.

Depending on the recurrence rate of shoulder dislocations, Habermeyer et al. [24] in a prospective study reported a torn or detached labrum following posttraumatic shoulder instability in 41–100% [24]. Furthermore, the authors found SLAP lesions in 16–33%, also depending on the recurrence rate of shoulder dislocations [24]. In this series, we found pathological findings of the capsule-ligament complex (including bony Bankart lesions, bony avulsions of the inferior glenoid and SLAP lesions) in 18%. Including patient number 7, who was suspected to have a Bankart lesion due to a subluxation of the humeral head seen on standard radiographs, the percentage rate rises up to 21%. As mentioned before, surgery allows better visualization and, therefore, more precise detection of accompanying pathologies. In contrast to Habermeyer et al. [24], who performed arthroscopy in all of their patients [24], we only performed surgery in six cases (16%). Therefore, it is rather likely that a few injuries following inferior shoulder dislocation might have been missed, especially regarding pathologies of the capsule-ligament complex.

Injuries of the axillary nerve following anteroinferior shoulder dislocation are rare (5–14%) and occur more often in older patients [25]. In this case series, we found axillary nerve palsy in 8%. Moreover, five more patients reported neurologic symptoms following inferior shoulder dislocation. A total of 21% of patients exhibited neurologic injuries. This higher rate of neurologic pathologies after LEH in comparison to the rate after anteroinferior shoulder dislocation might be explainable by the anatomical proximity of the brachial plexus, especially the axillary nerve to the inferior glenoid rim [26]. Although we are not able to prove this hypothesis in the present study, it seems probable that the brachial plexus is more likely to be injured when the humeral head is dislocated beneath the inferior glenoid rim. Interestingly, in two cases axillary nerve palsy was only noticed after reduction of the humeral head. However, several authors reported that injuries of the vascular or nervous system can occur either at the time of dislocation or reduction [15,27,28,29,30]. Nevertheless, with the exception of one case, neurologic symptoms dissolved in all our patients at the last follow-up. This finding is not surprising as it is known that axillary nerve palsy usually has a good prognosis due to the spontaneous healing of the nerve [31].

In contrast to the systematic review of Nambiar et al., who reported vascular injuries related to inferior shoulder dislocation in 10% of the cases, we did not observe any related vascular injuries in our series [2].

All patients received standardized radiographs, but only eight had a CT scan of the shoulder, although CT including a 3D-reconstruction model is crucial to determine bony defects on glenoid or humerus [32,33,34]. Furthermore, only nine patients obtained an MRI and only two patients underwent an MR-arthrography. MRI with or without the intraarticular application of a contrast agent is essential for the diagnosis of rotator cuff injuries and pathologies of the capsule-ligament complex. This lack of diagnostic measures raises the legitimate question whether all secondary pathologies regarding rotator cuff and capsule-ligament complex following inferior shoulder dislocation were detected in this series. Therefore, it seems essential to raise awareness of the high rate of associated shoulder injuries in case of inferior shoulder dislocation to minimize the probability of missing any secondary pathologies.

**Figure 5:**
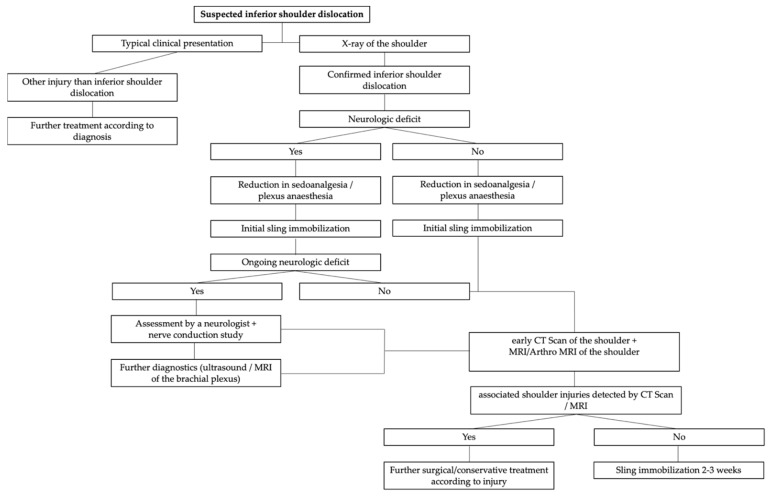
Management pathway based on the literature, study findings and authors’ experience.

As osseous injuries often occur after inferior shoulder dislocation (61% in our series), a CT scan should be performed with a low threshold. As LEH is further often associated with rotator cuff injuries (34% in our case series with a possibly high dark figure), an MRI should be performed as soon as possible to ensure a fast surgical treatment of eventual pathologies of the rotator cuff, since it is known that traumatic rotator cuff tears tend to degenerate rather quickly [17]. Moreover, it is crucial to examine the peripheral radial and ulnar pulses to ensure the vascular supply of the affected arm. Similarly, it is essential to check the neurologic integrity with special emphasis on the axillary nerve. If the neurologic examination is not conclusive or if neurologic integrity cannot be ensured, a determination of nerve conduction seems to be reasonable. A suggested management pathway based on the literature, study findings and authors’ experience is presented in Figure 5.

Limiting factors of the study are its retrospective design and obviously the fact that not every patient has been evaluated with the same diagnostic tools. The heterogeneity of diagnostics, especially the low number of performed CT scans and MRI, possibly lead to a high number of missed associated injuries. However, this is due to multiple reasons. First, patients included in this study have been seen over a period of more than 30 years and treatment strategies for shoulder dislocations have seen changes over the long study period. Secondly, due to the personal infrastructure of the two major trauma departments, there has been a great heterogeneity in physicians that treated the patients, with different physicians at most follow-up visits. Hence, there has been heterogeneity in treatment strategies and diagnostic pathways, with most of the physicians being trauma surgeons but not shoulder specialists. All these factors lead to the missing standardization of the investigations. However, reports of CT scans and MRI evaluations of inferior shoulder dislocations are very limited in the literature. Hence, we believe that our results might contribute to a better understanding and knowledge regarding a rare shoulder pathology, with little data available in the current literature.

The short follow-up period (mean 3 months) can be seen as another limiting factor; however, clinical follow-up was not the primary topic of this study. Special emphasis was applied on associated pathologies of inferior shoulder dislocation. The small number of patients included in the study can also be seen as a further limiting factor. However, we present the largest case series of inferior shoulder dislocation to date.

## 5. Conclusions

Inferior shoulder dislocation is a rare type of injury presenting with a high number of associated pathologies (82% in this series). According to the findings of this study, we want to point out and raise awareness of the high rate of potential secondary shoulder pathologies associated with LEH. Besides a thorough clinical examination and immediate standard anteroposterior and axial radiographs, we recommend to perform CT scans of the shoulder with a low threshold. To detect for potential rotator cuff injuries, an MRI with or without contrast agent of the shoulder should be conducted as soon as possible. In the case of neurologic deficiencies, a determination of nerve conduction should be performed.

## Figures and Tables

**Table 1:** Demographic data of included cases.

Patient	Sex	Age	Affected Side	Injury Pattern	Radiologic Examination	Method of Reduction	Immobilization (in Weeks)	Follow-Up (in Months)
1	M	15	Right	Volleyball	- Radiography - CT	Short anesthesia	3	1
2	M	16	Left	Handstand	- Radiography - CT - MRI	Short anesthesia	3	4
3	F	17	Right	Fall	Radiography	Conservative, without anesthesia	3	1
4	M	18	Right	Shadowboxing	Radiography	Conservative, without anesthesia	3	1
5	M	23	Right	Accident	- Radiography - US - MRI	Conservative, without anesthesia (from outside)	3	1
6	M	27	Right	Shoring up	Radiography	Interscalene block	3	1
7	M	28	Right	Sports	Radiography	Fluroscopy, without anesthesia	3	2
8	F	32	Left	Fall from ladder	- Radiography - MRI	Interscalene block	2	4
9	M	33	Left	Fall	Radiography	Short anesthesia	1	1
10	M	46	Left	Bicycle accident	- Radiography - MRI	Short anesthesia	3	5
11	M	48	Right	Fall	- Radiography - CT	Conservative, without anesthesia	3	1
12	M	49	Right	Fall	- Radiography - MRI	Short anesthesia	3	1
13	F	51	Left	Bicycle accident	- Radiography - CT - MR-arthrography	Conservative, without anesthesia	3	4
14	M	56	Right	Fall	Radiography	Conservative, without anesthesia	3	2
15	M	58	Right	Fall	Radiography	Conservative, without anesthesia	3	2
16	M	60	Right	Fall	- Radiography - MR-arthrography	Conservative, without anesthesia	3	11
17	M	63	Left	Tennis	- Radiography - MRI	Conservative, without anesthesia	2	4
18	M	64	Left	Bicycle accident	Radiography	Short anesthesia	3	2
19	M	66	Left	Sports	- Radiography - CT	Interscalene block	3	4
20	F	69	Left	Fall	Radiography	Short anesthesia	5	4
21	M	69	Right	Fall	- Radiography - MRI	Short anesthesia	3	3
22	F	71	Left	Fall	Radiography	Fluroscopy, without anesthesia	1 ^1^	1
23	M	72	Right	Fall	- Radiography - CT	Conservative, without anesthesia	4	3
24	M	77	Right	Fall, alcohol intoxication	Radiography	Conservative, without anesthesia	4	4
25	F	77	Right	Fall	Radiography	Short anesthesia	3	3
26	M	77	Left	Fall	Radiography	Short anesthesia	3	5
27	F	79	Left	Fall	Radiography	Conservative, without anesthesia	1 ^2^	3
28	F	86	Right	Fall	Radiography	Conservative, without anesthesia	2	1
29	F	87	Right	Fall	- Radiography - CT	Short anesthesia	1	1
30	F	90	Right	Fall	Radiography	Conservative, without anesthesia	3	1
31	F	91	Right	Fall	Radiography	Conservative, without anesthesia	3	1
32	M	20	Right	Fall	Radiography	Conservative, without anesthesia	3	1
33	F	67	Right	Fall	Radiography	Conservative, without anesthesia	3	2
34	M	63	Left	Fall	Radiography	Conservative, with anesthesia	4	8
35	M	40	Right	Sport	Radiography	Conservative, with anesthesia	3	2
36	M	29	Right	Sport	Radiography	Conservative, with anesthesia	3	3
37	W	68	Left	Fall	Radiography	Conservative, without anesthesia	3	1
38	M	57	Right	Fall, Epilepsia	Radiography	Operative treatment	3	25

CT = computed tomography; F = female; M = male; MRI = magnetic resonance imaging, US = ultrasound; ^1^ The patient was too disorientated for adequate immobilization; ^2^ The patient received a primary reverse shoulder prosthesis within the first week after LEH.

**Table 2:** Associated pathologies.

Patient	Bony Injuries	Rotator Cuff and LHBT Injuries	Capsule and Ligament Injuries	Neurologic Injuries	Further Surgical Intervention
1	None	None	None	None	None
2	- Bony Bankart lesion - Greater tuberosity fracture - Hill-Sachs lesion	None	Bony Bankart lesion	None	Arthroscopic Bankart repair
3	None	None	None	None	None
4	None	None	None	None	None
5	Bony avulsion of supraspinatus and infraspinatus tendon	Bony avulsion of supraspinatus and infraspinatus tendon	None	None	None
6	ALPSA lesion with glenoid bone loss (chronic luxations)	None	ALPSA lesion with glenoid bone loss	Mixed, not relatable (numbness in all fingers) ^1^	Open Latarjet procedure
7	None	None	Subluxation of humeral head in standard radiographs, suspected Bankart lesion	Ulnar nerve palsy (4th and 5th finger) ^1^	None
8	- Bony Bankart lesion - Hill-Sachs lesion	None	Bony Bankart lesion	None	None
9	Greater tuberosity fracture	None	None	None	None
10	Greater tuberosity fracture	None	None	None	None
11	None	None	None	None	None
12	Greater tuberosity fracture	None	None	None	None
13	None	Partial rupture of supraspinatus and infraspinatus tendon	SLAP III lesion	None	None
14	None	None	None	None	None
15	Greater tuberosity fracture	Restricted ROM, highly suspectable of rotator cuff lesion	None	None	None
16	None	- Rupture of the supraspinatus - tendon - Partial rupture of the LHBT	None	Axillary nerve palsy, after reduction ^1^	Arthroscopic rotator cuff repair, subacromial decompression and biceps tenodesis
17	Hill-Sachs lesion	- Rupture of the supraspinatus, - infraspinatus and subscapularis - tendon	- SLAP III lesion - Avulsion of posterior and inferior labrum	None	Arthroscopic rotator cuff repair, subacromial decompression and biceps tenotomy
18	Greater tuberosity fracture	None	None	None	None
19	Greater tuberosity fracture	None	None	None	None
20	Greater tuberosity fracture	None	None	None	None
21	None	- Rupture of the supraspinatus and subscapularis tendon - Partial rupture of the infraspinatustendon - Rupture of the LHBT	- SLAP III lesion	None	None
22	Greater tuberosity fracture	None	None	None	None
23	Greater tuberosity fracture	Restricted ROM, highly suspectable of rotator cuff lesion	None	Mixed, not relatable (numbness in 2nd, 3rd and 4th finger), after reduction ^1^	None
24	Bony avulsion of inferior glenoid	Proximal migration of humeral head in standard radiographs, highly suspectable of rotator cuff lesion	Bony avulsion of inferior glenoid	None	None
25	Greater tuberosity fracture	Restricted ROM, highly suspectable of rotator cuff lesion	None	Mixed, not relatable (numbness in all fingers) ^1^	None
26	Greater tuberosity fracture	Restricted ROM, highly suspectable of rotator cuff lesion	None	None	None
27	Proximal humeral head fracture with dislocation into the axilla	Total rupture of rotator cuff	None	None	Reverse total shoulder arthroplasty
28	None	None	None	None	None
29	Bony avulsion of inferior glenoid	None	Bony avulsion of inferior glenoid	Axillary nerve palsy	None
30	None	Restricted ROM, highly suspectable of rotator cuff lesion	None	None	None
31	None	Proximal migration of humeral head in standard radiographs, highly suspectable of rotator cuff lesion	None	Axillary nerve palsy, after reduction ^1^	None
32	None	None	SLAP III lesion	None	Outside hospital
33	None	None	None	Radialis palsy	None
34	None	None	None	None	None
35	posterior Hill-Sachs Lesion	Lesion of the LHBT	Labrum lesion anterior inferior	None	None
36	Greater tuberosity fracture	None	None	None	None
37	posterior Hill-Sachs Lesion	None	None	None	None
38	Impression of the humeral head	None	None	None	None

ALPSA = anterior labral periosteal sleeve avulsion; LHBT = long head of the biceps tendon; SLAP = superior labrum anterior and posterior; ROM = range of motion; ^1^ Neurologic pathologies dissolved at the latest follow-up.

**Table 3:** Summary of the key results regarding associated injuries.

Associated Shoulder Injuries (Patient Number/Percentage)	Associated Injuries to Other Body Regions (Patient Number/Percentage)	Associated Bony Shoulder Injuries (Patient Number/Percentage)	Associated Rotator Cuff/Tendon Injuries (Patient Number/Percentage); Numbers in Comma Include Suspected RC Injuries Due to X-ray or Clinical Findings	Associated Injuries to the Capsule-Ligament Complex (Patient Number/Percentage)	Associated Neurological Findings (Patient Number/Percentage)
31/82	6/16	23/61	7/18 (14/37)	7/18	8/21
		**Bony injuries broken down in total numbers**	**Rotator cuff/tendon injuries broken down in total numbers**	**Capsule-ligament complex injuries broken down in total numbers**	**Neurological findings broken down in total numbers**
		2 bony Bankart lesions 1 ALPSA lesion with bony flinge 2 bony avulsions of inferior glenoid 1 bony avulsion of rotator cuff 5 Hill-Sachs lesions 2 proximal humeral head FXs 13 FXs of the greater tuberosity	3 partial tears of SSP + ISP 4 complete SSP tears 1 complete ISP tear 2 complete SSC tear 1 total cuff tear 1 bony avulsion of the rotator cuff 1 partial rupture of the long head of the biceps 2 total ruptures of the long head of the biceps	1 avulsion of the posterior and inferior labrum 4 SLAP type III lesions 2 bony avulsions of the inferior glenoid 2 bony Bankart lesions 1 ALPSA lesion with bony flinge	1 ulnar nerve palsy 1 radial nerve palsy 3 axillary nerve palsies 3 mixed nerve palsies

Abbreviations: ALPSA, anterior labral periosteal sleeve avulsion; FX, fracture; SSP, Supraspinatus tendon; ISP, Infraspinatus tendon; SSC, Subscapularis tendon.

## Data Availability

The datasets generated and/or analyzed during the current study are not publicly available due to data privacy, but are available from the corresponding author upon reasonable request.
